# Current status and unanswered questions on the use of Denosumab in giant cell tumor of bone

**DOI:** 10.1186/s13569-016-0056-0

**Published:** 2016-09-14

**Authors:** Czar Louie Gaston, Robert J. Grimer, Michael Parry, Silvia Stacchiotti, Angelo Paolo Dei Tos, Hans Gelderblom, Stefano Ferrari, Giacomo G. Baldi, Robin L. Jones, Sant Chawla, Paolo Casali, Axel LeCesne, Jean-Yves Blay, Sander P. D. Dijkstra, David M. Thomas, Piotr Rutkowski

**Affiliations:** 1 Oncology Service, Royal Orthopaedic Hospital NHS Foundation Trust, Bristol Road South, Birmingham, B31 2AP UK; 2 Department of Cancer Medicine, Fondazione IRCCS Istituto Nazionale Tumori, Milan, Italy; 3 Department of Anatomic Pathology, General Hospital of Treviso, Treviso, Italy; 4 Leiden University Medical Center, Leiden, Netherlands; 5 Department of Oncology, Rizzoli Institute, Bologna, Italy; 6 Department of Cancer Medicine, S. Stefano Civil Hospital, Prato, Italy; 7 University of Washington/Fred Hutchinson Cancer Research Center, Seattle, WA USA; 8 Royal Marsden Hospital, London, UK; 9 Sarcoma Oncology Center, Santa Monica, CA USA; 10 Institut Gustave Roussy, Villejuif, France; 11 Centre Leon Berard, Lyon, France; 12 Garvan Institute of Medical Research, Sydney, Australia; 13 Department of Soft Tissue/Bone Sarcoma and Melanoma, Maria Sklodowska-Curie Memorial Center and Institute of Oncology, Warsaw, Poland

**Keywords:** Giant cell tumor of bone, Denosumab, Safety, Neoadjuvant, Inoperable, Surgery

## Abstract

Denosumab is a monoclonal antibody to RANK ligand approved for use in giant cell tumour (GCT) of bone. Due to its efficacy, Denosumab is recommended as the first option in inoperable or metastatic GCT. Denosumab has also been used pre-operatively to downstage tumours with large soft tissue extension to allow for less morbid surgery. The role of Denosumab for conventional limb GCT of bone is yet to be defined. Further studies are required to determine whether local recurrence rates will be decreased with the adjuvant use of Denosumab along with surgery. The long term use and toxicity of this agent is unknown as is the proportion of patients with primary or secondary resistance. It is advised that complicated cases of GCT requiring Denosumab treatment should be referred and followed up at expert centres. Collaborative studies involving further clinical trials and rigorous data collection are strongly recommended to identify the optimum use of this drug.

## Background

Giant cell tumour of bone (GCT) is a bone neoplasm which is locally aggressive and can rarely metastasize. Histone 3.3 mutations of the *H3F3A* gene were recently described for GCT of bone and may prove useful in clarifying diagnosis in challenging cases [1]. The incidence of GCT of bone has not been completely established but it is around half as common as osteosarcoma. This would suggest an incidence of around 1.5/million population per year. GCT typically arises at the end of a long bone in a skeletally mature individual but can also arise in the axial skeleton and occasionally in children. The standard treatment is surgery aiming for as near complete removal of the tumour as is possible without major morbidity. This is usually by detailed curettage, although in tumours with extensive bone destruction, resection or even amputation may be required. Adjuvant therapies during or after surgery such as the concomitant use of bone cement, phenol, ethanol, cryotherapy, or intravenous and oral bisphosphonates have been advocated to try and decrease the risk of local recurrence, but no randomised trial has ever been carried out to prove the efficacy of any of these [2–6]. Local recurrence is reported to occur in between 19–50 % of cases and usually arises within 2 years [4, 7–9]. Local recurrence can often be treated by repeat curettage but sometimes requires more morbid surgery to achieve complete tumour clearance.

GCTs with extensive soft tissue extension (grade 3 according to Campanacci grade 3 by radiology) and those of the axial skeleton are particularly challenging to treat and have been shown to have higher local recurrence rates [10–12]. In some cases, GCT of the spine and skull can be deemed inoperable, with complete tumour removal by surgery impossible due to the proximity of vital structures [12, 13]. Treatment in these difficult cases usually consists of debulking surgery (incomplete removal) and/or the use of adjuvants such as embolization [14, 15], radiation therapy [11, 16, 17], bisphosphonates [18, 19], and more recently, Denosumab [20, 21]. Conventional cytotoxic chemotherapy is not active in classic GCT of bone, even if anecdotal responses to osteosarcoma-like regimens containing platinum and anthracyclines have been reported in the metastastic setting [22–24].

### Denosumab

Denosumab is a fully humanised monoclonal antibody to RANK ligand (RANKL). Denosumab inhibits RANK-RANKL interaction, a key mediator of osteoclast activity, thereby resulting in reduction of osteoclast-induced bone destruction [25, 26]. Denosumab is currently approved by the FDA and European Medical Agency (EMA) for osteoporosis and also for prevention of skeletal related events in bone metastases from solid tumors.

Neoplastic stromal cells of GCT overexpress RANKL and activate osteoclast-like giant cells [27–29]. Denosumab treatment in GCT has been shown to reduce the number of tumour giant cells and neoplastic stromal cells and allow new bone formation [30]. The activity of Denosumab, dosed at 120 mg administered subcutaneously every 28 days with loading doses on days 8 and 15 (of the first month of therapy) was confirmed in a proof-of-principle phase II study on 37 GCT patients [25]. Tumor response was confirmed in 30 of 35 evaluable patients (86 %). Denosumab showed an improvement in Quality of Life (primary end point of the study) and good response in patients with inoperable GCT and reduced the need for otherwise morbid surgery in a second, larger phase 2 clinical trial [26]. Enrolled patients were separated into three cohorts: surgically unsalvageable GCT consisted mainly of patients with sacral and spinal GCT as well as metastatic pulmonary disease (cohort 1), patients who were planned for morbid surgery i.e. joint replacement, amputation, hemipelvectomy or major neurologic sequelae (e.g. base of skull tumours) (cohort 2) and those who transferred from the previous study of Denosumab (cohort 3). Cohort 1 showed no disease progression in 96 % (n = 163/169) of patients. 74 % of Cohort 2 (n = 74/100) in the trial did not need surgery while for those that did have surgery, 62 % (n = 16/26) required less morbid surgery than initially planned before Denosumab treatment. These data resulted in the FDA approval in June 2013 in adults and skeletally mature adolescents with giant cell tumour of bone deemed unresectable or requiring morbid surgery or in metastatic disease [31]. The EMA has also recently approved Denosumab for similar indications.

Administered as described above, Denosumab was generally well tolerated in trial patients but severe adverse events reported in the use of Denosumab include severe hypocalcaemia, osteonecrosis of the jaw (ONJ), and atypical stress fractures [26]. However, the cumulative and long term incidence of these toxicities remains to be accurately delineated and reported.

Since its introduction for use in GCT over 5 years ago, the reported cases treated with Denosumab has grown exponentially with at least 19 case reports and six case series being published in Pubmed from January to November 2015 alone. Whilst the FDA and EMA have specific criteria for Denosumab use in GCT of bone, clinical guidelines for use outside these criteria are lacking. Questions remain as to its recommended indications as well as the optimal duration of therapy. This is even more important as GCTB more often affects a young population of patients with a very long life expectancy. The potential of collaborative research projects to address these issues were discussed by the authors in a recent international meeting (November 2015) [32]. The results of those discussions are presented here.

### Current use of Denosumab in GCT of bone: indications and controversies

#### Locally advanced surgically unresectable and metastatic disease

**Fig. 1:**
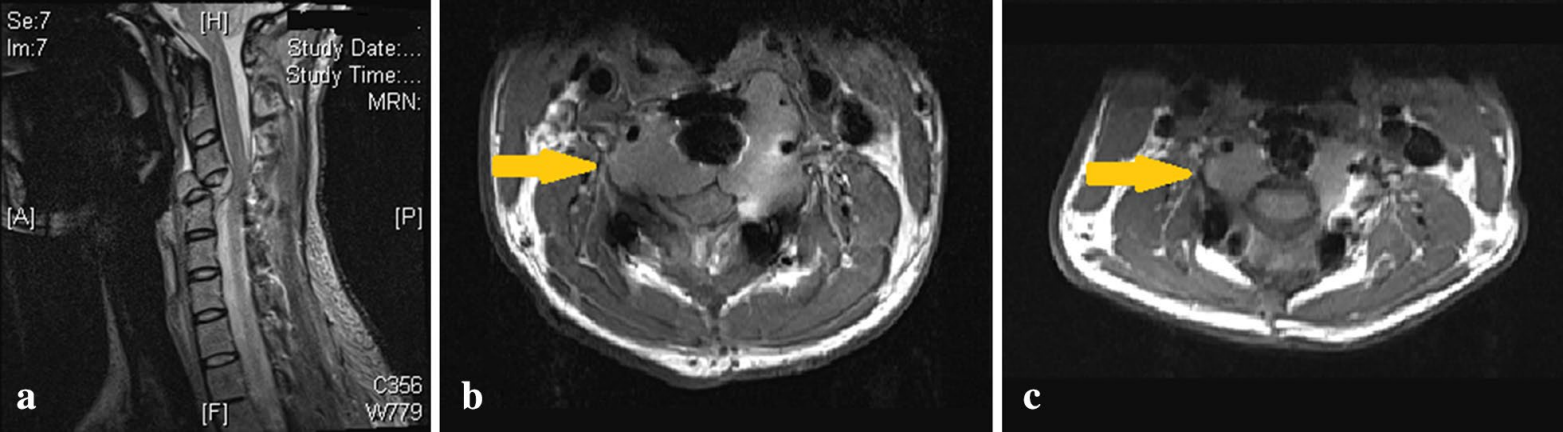
**a** Acute cervical cord compression secondary to collapse of giant cell tumour in C4 vertebrae. **b** 4 weeks from emergency decompression and stabilization, patient developed tumour recurrence (*arrow*) causing canal compression. **c** MRI scan after 3 months of Denosumab showing decrease in tumour mass (*arrow*) and decompression of spinal canal

The standard indication for Denosumab treatment is when there is no option of complete surgical removal of the tumour [26], such as tumours arising in the skull or spine or in the metastatic setting. Mattei reported a case of a 22 year old female with GCT of the C2 vertebral body and odontoid process successfully treated with Denosumab with a 16-month follow-up [21]. Recurrent GCT of the cervical spine after curettage and reconstruction presents a scenario without a surgical solution (Fig. 1). Treatment with Denosumab was associated with prompt pain relief and resolution of neurology. Denosumab has also been used successfully for control of metastatic lung disease [33–36].

Controversy exists however on how long treatment should be continued and what the long-term effects of such treatment may be. There is major concern that Denosumab withdrawal is associated with a high rate of subsequent tumour recurrence [37, 38] suggesting that Denosumab alone is insufficient to achieve pathological complete response. There is currently insufficient data to quantify this risk but many recurrences will arise within 7–9 months of stopping treatment. It is also not clear whether a rechallenge of Denosumab in case of secondary progression can achieve a new response. Mak et al. [37] studied GCT cell cultures and found that although Denosumab treated specimens did not show any giant cells, neoplastic stromal cells persisted and continued to proliferate albeit at a slower rate than untreated GCT. This may be explained by Lau et al.’s [38] findings that Denosumab caused only minimal inhibitory effects on GCT stromal cell lines and did not cause any apoptosis. Girolami et al. [39] found *H3F3A* mutation in pre-treatment and post-treatment surgical specimen of GCT of bone, supporting the hypothesis that the drug does not eliminate tumour cells.

It is therefore critical to rigorously evaluate in a prospective clinical trial the risk of relapse when patients stop Denosumab. Furthermore analysis of the ongoing prospective phase II trial data is essential in this regard. If tumour recurrence is inevitable once Denosumab treatment is stopped, then patients with inoperable disease may either need to receive long term treatment to prevent tumour progression or reconsideration may be made to alternative, definitive therapies such as radiotherapy [16, 17]. In females who wish to conceive and become pregnant, this will be a major concern as there is evidence that Denosumab is associated with increased still birth and decreased growth in animal infant studies [40]. Females on Denosumab are thus advised to avoid pregnancy and take appropriate contraceptive precautions while on the drug.

The long term effects of prolonged treatment with Denosumab are unknown and skeletal events due to suppressed bone turnover, such as atypical stress fractures [41] and critical hypercalcemia [35, 42] are of concern. The risk of ONJ has been reported to increase with the number and length of duration of Denosumab infusions [43] and this is a major concern in a benign tumour affecting young patients with little expected mortality secondary to disease. Chawla et al. [44] also recently presented data showing the increasing risk of toxicity after 2 years of treatment. Sustained continued use of Denosumab may need to be supplemented with rest periods (or ‘drug holidays’) as described for long term bisphosphonate use to minimise risk of atypical stress fractures in appropriate patients. If the clinical decision is made to stop Denosumab in the setting of inoperable disease, consideration may be given to using bone turnover markers to guide when Denosumab is to be restarted to prevent progressive disease recurrence. Urinary *N*-telopeptide and serum *C*-telopeptide determination show rapid decrease in levels once Denosumab treatment is started [25]. Tartrate-resistant acid phosphatase 5b, a bone resorption marker secreted by osteoclasts has also been shown to correlate with osteoclast activity systemically [45]. These biomarkers could be used to indicate when Denosumab has washed out of the system and potentially herald tumour recurrence.

Another approach to minimising adverse events in patients requiring long term treatment may be decreasing dose frequency once a steady state (9–12 months on Denosumab or maybe even less) has been achieved. In a phase 2 clinical trial, Denosumab given at a 12 week dosing schedule did not sustain suppression of bone turnover markers as well as a 4 week dosing schedule [46]. However, these results were in patients who had not yet achieved a steady state on the drug, which could explain the drop off in activity. Agrawal et al. [20] have used Denosumab given every 3 months as a ‘maintenance’ dose after treatment of extensive spinal disease. Importantly, it is not yet clear whether alterations in dosing schedule or drug holidays affect the longevity of tumour response.

#### Neoadjuvant use in difficult, locally advanced Campanacci 3 tumours

GCT of the pelvis, sacrum, and spine are associated with high local recurrence rates and significant surgical morbidity with conventional surgical treatment [11, 12, 14, 15, 47]. There are some case reports on axial GCTs where neo-adjuvant treatment with Denosumab helped make surgery possible [20, 48, 49]. In addition to the benefit of reducing tumor volume, the potential of Denosumab to reduce blood loss from intralesional curettage of pelvic GCT is alluded to by Watanabe et al. [45]. A retrospective analysis of a single institution experience from Girolami et al. [39] documented a conversion of the neoplastic stromal cells to a fibrous matrix with decreased angiogenesis, which could explain the decreased vascularity of the treated tumours. In a sub-study of the phase II clinical trial assessing 222 patients for possible downstaging with denosumab for planned surgery [50], the majority of patients after surgery received adjuvant Denosumab for 6 months. Of the 116 patients who had surgery (with median postsurgical follow-up of only 13 months), local recurrence occurred in 17 (15 %) patients—the majority of these patients underwent local excision only.

Campanacci 3 lesions treated with curettage are associated with higher local recurrence rates versus Campanacci 1 or 2 lesions [4, 10]. In extensive grade 3 lesions, the extension of the tumour into the soft tissues as well as involvement of the articular cartilage usually means that an effective curettage is not always possible and these cases may be better treated with a resection of the involved bone. Denosumab treatment in GCT causes a rim of new bone to form [30, 51], which effectively converts what was previously a Campanacci 3 lesion to a lesser grade. With an increasing rim of ossified bone on the periphery, intralesional curettage is more possible [52].

There is concern however about the ability to do an effective extended curettage after Denosumab treatment. Although the newly formed bone on the periphery allows for a sufficient mechanical scaffold for curettage to be done without fear of the bone collapsing, the rim of new bone may contain neoplastic cells that may reactivate once Denosumab treatment is finished. In the phase 2 clinical trial, the median time to surgery after Denosumab treatment was around 2 years and after this length of time on the drug, the rim of new bone and ossification on the periphery of the tumour is quite thick, such that the probability of leaving behind neoplastic tissue would be quite high. In this situation, an adjuvant such as liquid nitrogen that penetrates throughout the ‘new bone’ that has formed may be beneficial [53]. Performing definitive surgery much earlier (3–4 months after starting Denosumab) to prevent too thick a rim of bone from forming may make complete removal much more feasible. There is also the possibility of keeping a patient on a maintenance dose of Denosumab to prevent recurrence [20]. Whether this will be sufficient to maintain long term control remains unknown. A comparative study between neo-adjuvant Denosumab and curettage versus outright resection for extensive Campanacci 3 tumours is needed to accurately determine long term disease control and functional outcomes.

A different situation exists when wide radical surgery is planned after Denosumab therapy for tumours that are very advanced locally with a large soft tissue mass, joint involvement, or pathological fracture where primary amputation or wide resection is required for complete tumour clearance. In these situations, reduction of tumour volume and calcification of the tumour penetrating into soft tissues after Denosumab therapy can facilitate or enable radical tumour resection. When en-bloc resection is planned neoadjuvant therapy should be used for longer time until maximal calcification of the tumor and response plateau is observed on consecutive imaging.

#### Progression and recurrence

The vast majority of patients on Denosumab will have clinical and radiological evidence of response, usually manifest by decrease in pain, increase in function and sometimes radiological shrinkage, often accompanied by a decrease in activity on PET scan and formation of calcification. Using conventional radiological criteria, response by RECIST which is based on decreases in tumour diameter do not adequately describe the therapeutic response to Denosumab due to minimal tumour shrinkage [54]. Using EORTC criteria, which incorporate changes in PET-FDG uptake, and inverse Choi, measuring increase in density with calcification of the tumour, Denosumab has shown good response for GCT of bone compatible with the clinical improvement seen in the majority of patients on the drug [26, 54]. Denosumab activity often results in osteosclerosis, calcified rim formation, and reconstitution of cortical bone often without significant changes in overall dimensions that can be detected on conventional radiographs and computed tomography. On dynamic contrast-enhanced magnetic resonance imaging (MRI), later enhancement followed by slower washout compared with index MRI may indicate response to treatment, usually the infiltration within soft tissues is decreased. The optimal radiologic tool for assessment is not yet known although MRI scans seem to be adequate to document response to treatment while CT scans may be used to monitor reconstitution of the cortical rim for surgical planning.

Data on GCT of bone patients resistant to Denosumab are scanty and the final analysis of the phase II study is awaited. The latter must include the incidence of complications from treatment as well as the rates of local recurrence in patients who have had surgical resection, be it a complete excision or curettage. Overall, available data show that progression while on treatment is unusual [26] and is usually associated with increase in pain and size of the lesion. In some situations part of a tumour may progress while other areas remain controlled and the mechanism of this is not clear. In tumours that do not respond to Denosumab from the beginning or after an initial response, both the original biopsy and recurrent tissue should be carefully reviewed to exclude malignant GCT or other giant-cell rich pathology. There are isolated reports of ‘benign’ GCTs recurring as ‘malignant’ following Denosumab treatment but the incidence of this is unknown [55]. Finally, molecular mechanisms of resistance to Denosumab in GCTB are still left to be fully understood.

In patients who have stopped Denosumab (for whatever reason) and develop recurrence, anectodal reports suggest that if clinically indicated, Denosumab can be effective at treating recurrence which the final results of the phase II trial should answer.

## Conclusions

Denosumab is an effective and useful drug for managing GCT of bone. It should be considered as the gold standard for first line treatment for patients with inoperable or metastatic GCT. The optimal treatment schedule in long term maintenance therapy with less frequent dosage is not known and should be the subject of ongoing research. Reintroduction of Denosumab following recurrence of GCT after stopping therapy for different reasons seems to be an effective option. Denosumab can be used to downstage those with disease requiring morbid surgery, but the timing of use of neoadjuvant therapy in locally advanced Campanacci grade 3 tumors is debatable. Further randomized studies are required to determine whether local recurrence rates will be decreased with the adjuvant use of Denosumab along with surgery. Fortunately, the numbers required to answer these questions seem to be achievable within reasonable timeframes, based on the successes of the initial and subsequent trials of Denosumab in GCT of bone [25, 26, 54]. Safety for long term use is unknown and should be reported as soon as possible with the full dataset of the large phase II study. Due to the challenges of treating this disease and the unanswered questions regarding optimal use of Denosumab, referral and follow-up of complicated cases of GCT requiring Denosumab should be within expert centres with a multidisciplinary team. Further clinical trials are mandated to identify the optimum indications for using Denosumab in GCT.
